# Epigenetic regulation of cardiac myocyte differentiation^†^

**DOI:** 10.3389/fgene.2014.00375

**Published:** 2014-11-04

**Authors:** Kyohei Oyama, Danny El-Nachef, Yiqiang Zhang, Patima Sdek, W. Robb MacLellan

**Affiliations:** Division of Cardiology, Department of Medicine, Center for Cardiovascular Biology and Institute for Stem Cell and Regenerative Medicine, University of WashingtonSeattle, WA, USA

**Keywords:** cardiac myocyte, proliferation, differentiation, heterochromatin, histone modification, retinoblastoma protein

## Abstract

Cardiac myocytes (CMs) proliferate robustly during fetal life but withdraw permanently from the cell cycle soon after birth and undergo terminal differentiation. This cell cycle exit is associated with the upregulation of a host of adult cardiac-specific genes. The vast majority of adult CMs (ACMs) do not reenter cell cycle even if subjected to mitogenic stimuli. The basis for this irreversible cell cycle exit is related to the stable silencing of cell cycle genes specifically involved in the progression of G2/M transition and cytokinesis. Studies have begun to clarify the molecular basis for this stable gene repression and have identified epigenetic and chromatin structural changes in this process. In this review, we summarize the current understanding of epigenetic regulation of CM cell cycle and cardiac-specific gene expression with a focus on histone modifications and the role of retinoblastoma family members.

## INTRODUCTION

The fetal heart increases in size throughout development via proliferation of CMs but switches to mainly hypertrophic growth of CMs with limited proliferation soon after birth, undergoing terminal differentiation which is associated with permanent cell cycle exit (Ahuja et al., 2007; Mollova et al., 2013; Naqvi et al., 2014). Terminal differentiation in ACMs is characterized by two distinct phenomena: the upregulation of a panel of cardiac-specific adult genes and the permanent withdrawal from cell cycle (Ahuja et al., 2007). The inability of ACMs to proliferate has been linked to the fact that E2F-dependent cell cycle genes specifically involved in regulating G2/M and cytokinesis are not re-expressed after growth stimuli in ACMs. Recent studies suggest the upregulation of adult cardiac-specific genes together with the silencing of cell cycle genes may be mediated by epigenetic mechanisms (Sdek et al., 2011).

Epigenetic mechanisms regulate chromatin structure (Li and Reinberg, 2011), which modulates gene expression and plays a crucial role in diverse biological events such as the specification and differentiation of various cell types (Chen and Dent, 2014). Epigenetic marks have traditionally been thought to be stable, however the recent identification of histone modification enzymes suggests that epigenetic regulation can be a dynamic and reversible process (Kooistra and Helin, 2012). This review focuses on epigenetic regulation in CMs and its role in cell cycle control and terminal differentiation with a focus on histone modifications and Rb family members.

## EPIGENETIC REGULATION OF GENE EXPRESSION AND SILENCING

Epigenetics is typically defined as the regulatory mechanisms of gene activity that are not due to changes in DNA sequence. These include modifications of DNA and histone proteins, which affect chromatin structure, and microRNA. In the nuclei of eukaryotic cells, DNA is wrapped around an octamer of histone proteins that are packed into higher-order chromatin structures. Epigenetic regulation involves covalent modification of either of DNA (DNA methylation) or of nucleosomes, which is primarily through post-translational modification of histones (acetylation, methylation, phosphorylation, ubiquitination, sumoylation, ribosylation, deamination, and proline isomerization; Chen and Dent, 2014). These epigenetic post-translational modifications are tightly controlled by specific enzymes, for example HATs and HDACs as well as HMTs and HDMs. There are two fundamental types of chromatin: euchromatin and heterochromatin. Euchromatin is typically associated with transcriptionally active genes because its looser structure is accessible to TFs. In contrast, heterochromatin has a high-density structure that prevents transcriptional machinery access and gene expression (Johnson et al., 2013).

Euchromatin formation is typically associated with histone acetylation, on the other hand, heterochromatin formation is associated with specific histone methylations. The effect of histone methylation is dependent on which amino acid residue of the histone is methylated, and whether the residue is mono, di, or trimethylated (me1, me2, and me3, respectively; Chen and Dent, 2014). For example, methylation of the lysine 4, 36, or 79 lysine residue of histone 3 (H3K4me, H3K36me, and H3K79me) at gene promoters is associated with transcription activation, while methylation of the 9th or 27th lysines (H3K9me, H3K27me) is linked to heterochromatin formation and gene repression (Chen and Dent, 2014). H3K9me3 is a potent inducer of stable heterochromatin by recruiting HP1s (Canzio et al., 2013). H3K27me3 is thought to be more dynamically regulated and mark repressed but poised genes (Rada-Iglesias et al., 2011; Lee et al., 2012). The addition of methyl groups on the lysine and arginine residues of histones is catalyzed by HMTs, while the removal of methyl groups is mediated by HDMs. For all the known lysine residues of HMT activity, counteracting HDMs have been identified, with the exception that the H3K79 HDM is not known, although evidence suggests methylations of this mark are reversible (Kooistra and Helin, 2012).

## EPIGENETIC REGULATION OF CARDIAC-SPECIFIC GENE EXPRESSION

During cardiac differentiation, the epigenetic landscape changes dramatically, which is required for appropriate cardiac differentiation (Paige et al., 2012; Wamstad et al., 2012). The establishment and maintenance of a specific gene expression program includes activation of cardiac-specific genes and cell cycle inhibitors as well as stable repression of non-cardiac genes and cell cycle progression genes.

### HATs, HDACs, AND HISTONE ACETYLATION

**FIGURE 1:**
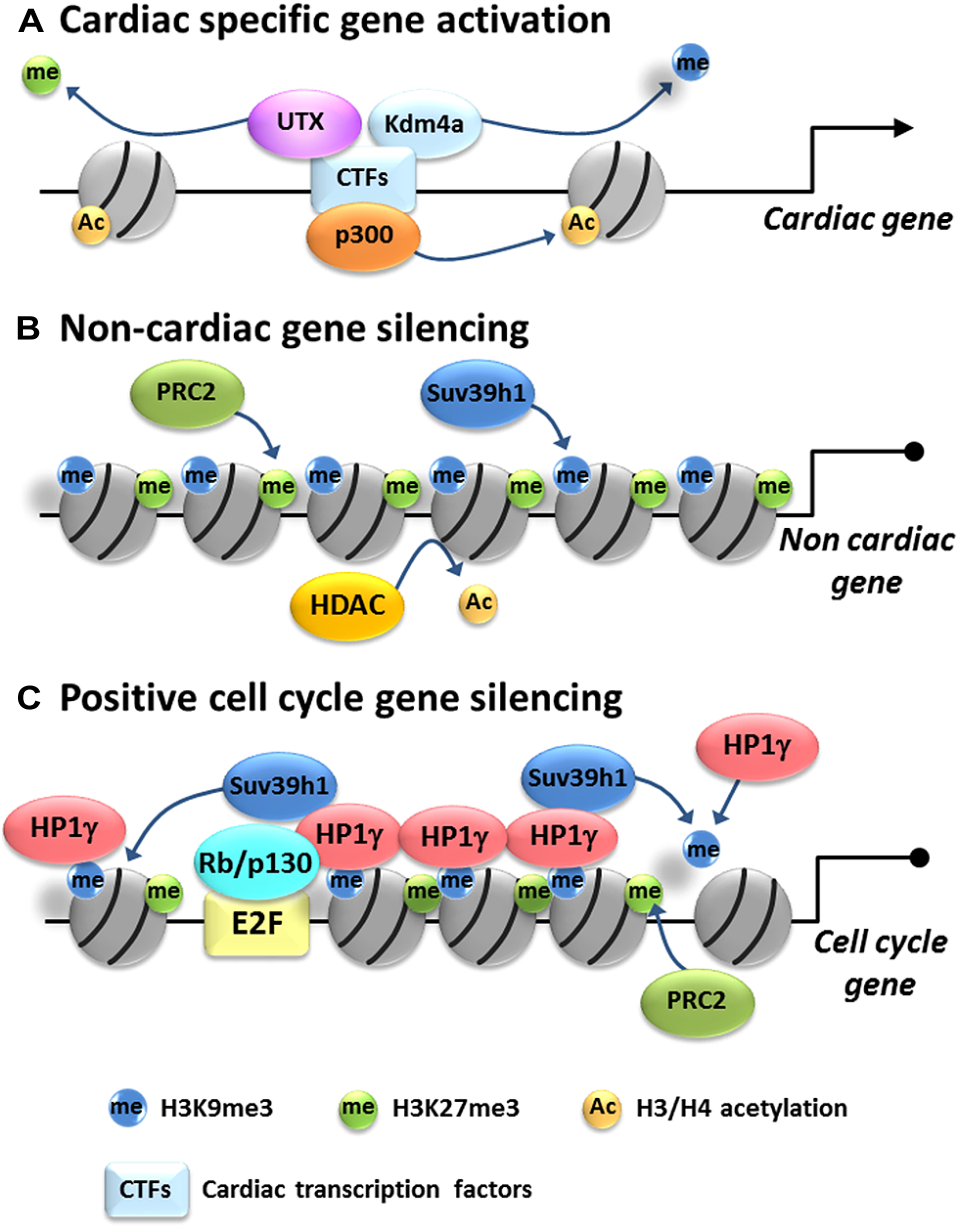
**Model of epigenetic gene regulation in cardiac myocytes.** Cardiac differentiation is associated with activation of cardiac-specific genes and silencing of non-cardiac and cell cycle genes. **(A)** Cardiac-specific gene regulation: cardiac-specific transcription factors (CTFs) recruit histone acetyltransferases, such as p300, transferring acetyl groups to histone H3 and/or H4. Also they recruit histone demethylases such Kdm4a and UTX to remove silencing methyl marks from H3K9me3 and H3K27me3, resulting in activation of cardiac-specific genes. **(B)** Non-cardiac gene repression: HDACs remove acetyl groups from H3 and/or H4 and histone methyltransferases such as Suv39h1 and PRC2 put methyl groups on H3K9 and H3K27, respectively, promoting tighter histone packing and silencing non-cardiac genes. **(C)** Cell cycle gene silencing: Rb/E2F complex targets HP1γ on positive cell cycle gene promoters. HP1γ spreads H3K9me3 likely through recruitment of Suv39h1 and self-assembles to condensate chromatin, resulting in the packaging and silencing of positive cell cycle genes in heterochromatin. H3K27me3 is also enriched by an unknown mechanism but probably mediated by PRC2.

The HAT most studied in cardiac development is p300. p300 is highly expressed in embryonic myocardium but the level declines after birth (Schueler et al., 2012). 3,000–5,000 potential enhancers are associated with p300 in fetal and adult hearts (Blow et al., 2010; May et al., 2012), suggesting an important role in CM development. p300-deficient mice are embryonic lethal at E9-11.5 with heart malformations and reduced expression of cardiac-specific genes such as αMHC and αSA (Yao et al., 1998; Partanen et al., 1999). Knock-in experiments using an acetyltransferase activity-deficient p300 mutant demonstrated that p300 acetyltransferase activity is specifically required for cardiac development (Shikama et al., 2003). p300 interacts with GATA4, Nkx2.5, and Mef2c, which are key TFs regulating CM gene expression and differentiation, at promoters of their target genes (Sun et al., 2010; **Figure 1A**). In addition, expression of cardiac-specific genes such as atrial natriuretic peptide (ANP) and brain natriuretic peptide (BNP) correlates with p300 occupancy and histone acetylation on their promoters (Hasegawa et al., 1997; Slepak et al., 2001; Mathiyalagan et al., 2010; Schlesinger et al., 2011; Schueler et al., 2012). Consistent with this, inhibition of p300 resulted in decreased expression of cardiac-specific genes such as α-MHC and β-MHC and interestingly the expression of cardiac TFs such as Mef2c, Nkx2.5, and Hands were also decreased (Hasegawa et al., 1997; Lin et al., 1997; McFadden et al., 2000; Poizat et al., 2000; Dai et al., 2002). Cardiac TFs such as GATA4 and Mef2c can be directly acetylated by p300 and the acetylation potentiates DNA binding and transcriptional activity (Kawamura et al., 2005; Ma et al., 2005). CBP, a HAT structurally related to p300, is expressed in embryonic hearts, but CBP-deficient embryos don’t show abnormal heart formation (Tanaka et al., 2000; Chen et al., 2009). Males absent on the first (MOF) protein, a HAT belonging to the MYST (MOZ, Ybf2/Sas3, Sas2, and TIP60) family member, is down-regulated in human failing hearts and mouse hypertrophic hearts (Qiao et al., 2014). Cardiac-specific MOF overexpression ameliorated TAC-induced cardiac hypertrophy, however it was not determined if this protection was related to HAT activity or targeting of specific genes (Qiao et al., 2014). Other HATs such as Gcn5 have been implicated in cardiac differentiation *in vitro* but their relative importance is unknown (Li et al., 2010).

The effects of HATs are counteracted by HDACs, which typically repress gene activation. HDACi TSA promotes acetylation of H3 and H4 and CM differentiation *in vitro* (Kawamura et al., 2005; Karamboulas et al., 2006). Cardiac-specific deletion of either HDAC1 or HDAC2 singly does not evoke a phenotype; however, deletion of both genes results in neonatal lethality, accompanied by cardiac arrhythmias and dilated cardiomyopathy (Montgomery et al., 2007). Mice with cardiac-specific overexpression of HDAC3 show a decrease in global H4 acetylation and an increased thickness of myocardium which is due to cardiac hyperplasia without hypertrophy (Trivedi et al., 2008). The hyperplasia is related to suppression of Cdk inhibitors such as p21^cip1^, p27^kip1^, p57^kip2^, p18^inc4c^, and p15^inc4b^. In contrast, mice with a cardiac-specific deletion of HDAC3 survived up to 4 months of age but demonstrated massive cardiac hypertrophy, myocardial lipid accumulation and elevated triglyceride levels (Montgomery et al., 2008). Indeed, ChIP assays show that HDAC3 co-occupies promoters of numerous genes involved in metabolic regulation with PPARα. It seems that HDAC3 is an important regulator of CM proliferation and energy metabolism during cardiac development. Interestingly global histone acetylation is unchanged in HDAC3 KO mice, suggesting that the effects of HDAC3 deficiency are very specific. HDAC4, a class II HDAC, has an anti-hypertrophic role through Mef2 suppression. Recent studies suggested that HDAC4 suppresses Mef2 in a histone deacetylation activity independent manner (Backs et al., 2011; Hohl et al., 2013). Indeed histone acetylation did not change on hypertrophic gene promoters when HDAC4 nuclear activity was reduced (Hohl et al., 2013). HDAC5 and HDAC9 are highly enriched in the heart and their functions are overlapping during cardiac development (Haberland et al., 2009). Single HDAC5 or HDAC9 KO mice are viable without apparent cardiac defects but mice lacking both HDAC5 and HDAC9 are embryonic or early postnatal lethal with ventricular septal defects, thin-walled myocardium and abnormality of CMs (Zhang et al., 2002; Chang et al., 2004). Since HDAC5 and HDAC9 interact with Mef2 to suppress its transcriptional activity (Zhang et al., 2002; Chang et al., 2004), the developmental cardiac defects in the double mutant mice are likely resulted from aberrant activation of Mef2. Interestingly it has been shown recently that HDAC can also be acetylated during cardiac hypertrophy, which alters their function (Eom et al., 2014). Numerous reports using inhibitors and gene manipulation techniques have revealed the importance of HAT/HDAC in cardiac development. However, the specific target genes and the histone acetylation-independent mechanism of each HAT/HDAC and their roles in cardiac development require further study.

### HMTs, HDMs, AND HISTONE METHYLATION

There is also increasing evidence demonstrating the importance of histone methylation in regulating cardiac phenotypes (Gottlieb et al., 2002; Barski et al., 2007; Nimura et al., 2009; Fujii et al., 2011; Movassagh et al., 2011; Tao et al., 2011; He et al., 2012a; Lee et al., 2012). Recent exome sequencing analysis revealed that congenital heart disease cases show a marked excess of *de novo* mutations in genes involved in H3K4 and H3K27 modifications (Zaidi et al., 2013). ACMs with inducible, cardiac-specific KO of H3K4 HMT subunit, PAX interacting (with transcription-activation domain) protein 1 (PTIP), showed altered expression of genes involved in conduction, such as Kcnip2, but not genes involved in hypertrophy, such as β-MHC and ANP (Stein et al., 2011). Specific deletion of PTIP in ACMs led to dysregulated sodium and calcium handling, abnormal EKGs, and susceptibility to ventricular premature beats, but no abnormalities of cardiac growth. Smyd1 is a cardiac and skeletal muscle restricted chromatin remodeling protein that can also methylate H3K4 *in vitro*, suggesting it may function as a muscle-specific transcription activator (Sims et al., 2002; Tan et al., 2006; Sirinupong et al., 2010). Smyd1 deficient mice die *in utero* secondary to abnormal CMs maturation and right ventricular development (Gottlieb et al., 2002). Consistent with the right ventricular development defect, the expressions of Hand2 and Irx4 are downregulated in hearts lacking Smyd1 (Gottlieb et al., 2002; Park et al., 2010). Muscle-specific TF skNAC is a major partner for Smyd1 in the developing heart (Park et al., 2010; Sirinupong et al., 2010) and normal expression of Hand1 and Irx4 is dependent on Smyd1-skNAC interaction (Park et al., 2010). It is not clear if the defect in cardiac development and cardiac-specific gene expression in Symd1 deleted mouse is directly related to its HMTase activity (Tan et al., 2006; Just et al., 2011). Interestingly Symd1 interacts with sarcomere protein and potentially methylates myosin protein (Just et al., 2011; Li et al., 2013). Symd1 can also function as a transcriptional repressor by recruiting class I HDAC (Gottlieb et al., 2002; Costantini et al., 2005).

Another Smyd family member, Smyd2, is a H3K4 and H3K36 HMT that is highly expressed in neonatal CMs. A CM-specific KO of Smyd2 showed it is dispensable for normal cardiac development and had no effect on H3K4 and H3K36 methylation in mice, perhaps due to redundant HMTs that can compensate for the Smyd2 deficient (Diehl et al., 2010). Wolf-WHSC1 is a H3K36 HMT, which catalyzes mono-, di-, and tri-methylation. Deletion of WHSC1 is observed in all patients with Wolf–Hirschhorn Syndrome, which is associated with cardiac congenital defects (Bergemann et al., 2005). WHSC1 KO mice die perinatally with atrial and ventricular septal defects (Nimura et al., 2009). WHSC1 interacts with Nkx2.5 and occupies Nkx2.5 target genes to repress transcription presumably through H3K36me3 modification. Distinct H3K36me methylation patterns have been described for end-stage cardiomyopathic compared to age-matched normal human hearts (Movassagh et al., 2011), suggesting proper regulation of H3K36me may be important for cardiac development and maintaining physiological ACMs gene expression in humans as well.

Histone methyltransferases have also been implicated in cardiac health and disease. PRC2 is a HMT complex, which consists of four components: catalytic subunit enhancer of Zeste 1 (Ezh1)/Ezh2, suppressor of Zeste 12 (Suz12), embryonic ectoderm development (Eed), and RbAp46/48 (Margueron and Reinberg, 2011). PRC2 mediates the methylation of H3K27, which silences genes and regulates tissue-specific differentiation by orchestrating the repression of unnecessary or stage-specific transcriptional programs (Boyer et al., 2006; Pasini et al., 2007; Shen et al., 2008). During CM development, H3K27me3 levels increase when cardiac progenitor cells are differentiating into CMs (Delgado-Olguin et al., 2012). In the heart, Ezh1 and Ezh2 are predominantly expressed in adult and embryonic stage, respectively (Sdek et al., 2011). The importance of PRC2 in cardiac development has been demonstrated using cardiac-specific deletion models (Chen et al., 2012; Delgado-Olguin et al., 2012; He et al., 2012a). Conditional inactivation of Ezh2 specifically in right ventricle progenitors by Mef2cAHF-Cre, which is active from E7.5, caused right ventricle hypertrophy (Delgado-Olguin et al., 2012). This hypertrophy was caused by derepression of Six1 gene which is stably silenced upon cardiac differentiation. Nkx2.5-Cre driven Ezh2 inactivation in early cardiac differentiation caused embryonic lethality with defects in heart development; however, inactivation of Ezh2 in differentiated CMs by TnT-Cre did not evoke a phenotype (He et al., 2012a). The developmental defect in Ezh2 deficient mice was associated with aberrant expression of non-cardiac and cell cycle inhibitor genes and ectopic expression of atrial-specific genes in ventricular myocytes. These findings indicate that Ezh2 and H3K27me3 promote and stabilize cardiac differentiation by silencing ectopic gene programs. Interestingly, Ezh2 directly binds to GATA4 and also methylates it, which attenuates its transcriptional activity by reducing its interaction with p300. This interact is important for suppression of αMHC expression in embryonic CMs (He et al., 2012b). G9a and GLP are major H3K9 mono- and dimethyltransferases and contribute to transcriptional silencing. Nkx2.5-Cre driven dual function loss of GLP and G9a (GLP-KO/G9a-KD) mice showed reduction of H3K9me2 level in CM and atrioventricular septal defects, but not in single either GLP or G9a function loss (Inagawa et al., 2013). Array analysis revealed expression of non-CM gene in GLP-KO/G9a-KD CM. Suv39h1 which mediates tri-methylation of H3K9 can regulate cell cycle exit in cardiac differentiation (Sdek et al., 2011). Silencing of Suv39h1 in ACMs by siRNA increased the expression of cell cycle progression genes and consistent with this silencing of HP1γ which binds H3K9me3, also induced cell cycle progression gene upregulation (Sdek et al., 2011). Thus deposition of suppressive histone marks such as H3K9me2/3 and H3K27me3 seems to be involved in non-cardiac gene silencing and cell cycle exit (**Figure 1B**).

Jumonji, coded by the Jarid2 gene, is a nuclear factor that plays an essential role in the development of multiple tissues, including the heart. Jmj has a DNA binding domain, ARID, and two conserved Jmj domains (JmjN and JmjC; Takeuchi et al., 2006). Based on homology to the Jmj domain, it is now recognized that this protein is part a family of proteins, most of which are associated with histone modifying activity. The JmjC domain is essential for the demethylase activity (Klose et al., 2006; Takeuchi et al., 2006). The identification that the Jmj family proteins have HDM activity, which can demethylate mono-, di and tri-methylation, suggested that histone methylation might be more dynamic than previously thought and regulate acute changes in gene expression (Bose et al., 2004; Tsukada et al., 2006; Lan et al., 2008; Lee et al., 2012). The Jmj family member Jmjd6 is a histone H3 and H4 arginine demethylase that is essential for cardiac development (Chang et al., 2007). Jmjd6-deficient mice die perinatally due to cardiac malformations with ventricular septal defect and double-outlet right centicle (Schneider et al., 2004). Kdm4a is a H3K9me3 and H3K36me3 HDM (Whetstine et al., 2006). In failing hearts ANP and BNP promoters have less enrichment of H3K9me3 (Hohl et al., 2013). Consistent with this Kdm4a is upregulated and enriched on these promoters (Zhang et al., 2011; Hohl et al., 2013). Although cardiac-specific Kdm4a deficient mice and transgenic mice which overexpress Kdm4a show no overt baseline phenotype (Zhang et al., 2011), when subjected to pressure overload, inactivation of Kdm4a attenuates hypertrophic response while Kdm4a overexpression enhances cardiac hypertrophy (Zhang et al., 2011). This Kdm4a mediated hypertrophy can be related to the expression of FHL1, a key component of the mechanotransducer machinery which is involved in hypertrophic development (Sheikh et al., 2008), via demethylation of H3K9me3 on FHL1 promoter. Recently another JmjC protein ubiquitously transcribed tetratricopeptide repeat, X chromosome (UTX), a H3K27 demethylase encoded on X chromosome, was shown to be essential for cardiogenesis (Agger et al., 2007; Hong et al., 2007; Lan et al., 2007). UTX is highly expressed in developing hearts and its deletion in female mice is embryonic lethal with severe cardiac malformation (Lee et al., 2012). UTX null embryonic stem cells (ESCs) also fail to develop spontaneous contractions and cardiac-specific gene expression (ANP, MLC2, and a-CA). UTX interacts with core cardiac TFs, Nkx2.5, Tbx5, GATA4, and serum response factor (SRF) as well as cardiac-specific enhancer of Brg1-associated factor Baf60c and potentiates their transcriptional activity to activate cardiac-specific genes (Lee et al., 2012). Interestingly, in addition to H3K27me3, UTX can also affect H3K4 methylation for activation of cardiac enhancers (Lee et al., 2012). This is likely to be an indirect effect of the loss of UTX due to the fact that UTX and MLL3/4 are in the same complex. Thus removal of silencing histone marks is important for cardiac-specific gene activation.

## EPIGENETIC REGULATION OF CM TERMINAL DIFFERENTIATION

Terminal differentiation is not the only situation under which adult cells become postmitotic. Senescent cells also undergo an irreversible cell cycle arrest. In both situations, cells are unable to express the genes required for proliferation, even when stimulated with growth factors. At the molecular level, nuclei of senescent and terminally differentiated cells demonstrate accumulation of heterochromatin. This heterochromatin is a characteristic feature of the irreversible cell cycle exit of senescent and terminally differentiated cells (Narita et al., 2003; Brero et al., 2005; Sdek et al., 2011, 2013). Large-scale chromatin condensation and the reorganization of nuclear domains reduce the accessibility of transcription machinery within heterochromatic loci (Grewal and Jia, 2007). Localization of E2F target genes in heterochromatin regions is seen in both senescent and terminally differentiated cells (Narita et al., 2003; Sdek et al., 2011). Heterochromatic regions are characterized by histone hypoacetylation and enrichment of H3K9me3. Chromatin of proliferating embryonic CMs is hyperacetylated (H3K9/14, H3K18, and H3K27), but following adult differentiation, acetylation deceases and histone methylation (H3K9me3 and H3K27me3) associated with transcriptional repression predominates (Sdek et al., 2011).

### HISTONE ACETYLATION

Histone deacetylation mediated by HDACs is the initial step of heterochromatin assembly. Although little is known about the function of HDACs in CMs terminal differentiation, among the over 18 HDAC family members, HDAC1 plays critical role in regulation of proliferation in other cell types; however, the effects of HDAC1 on cell cycle are developmentally dependent. Deletion of HDAC1 results in embryonic lethality at E9.5 due to impaired cellular proliferation (Lagger et al., 2002). However, in cellular senescence, HDAC1 promotes irreversible silencing of proliferation related genes (Stadler et al., 2005; Bandyopadhyay et al., 2007; Willis-Martinez et al., 2010; Chuang and Hung, 2011). HDAC1 and hypo-phosphorylated Rb protein levels are elevated in senescent cells (Narita et al., 2003; Wang et al., 2008). HDAC1 forms a complex with Rb and E2F4 on the E2F-dependent promoters and is responsible for deacetylating histone H3 on E2F-dependent promoters (Wang et al., 2008). A critical role for HDAC1 in terminal differentiation has been revealed in several cell types (Stadler et al., 2005; Yamaguchi et al., 2005; Ye et al., 2009). HDAC1 is required for the switch from proliferation to differentiation by antagonizing Wnt and Notch signaling pathways to promote cell-cycle exit and the subsequent neurogenesis in zebrafish retina (Yamaguchi et al., 2005). Loss of HDAC1 in retina results in failure of differentiation, which correlated well with failure of precursor cells exit cell cycle and upregulation of proliferation promoting proteins (Stadler et al., 2005). An inducible-cardiac-specific model to delete HDACs at different stages in differentiating CMs might shed light on the specific roles of HDACs in CMs differentiation.

### H3K9 METHYLATION

Di- and tri-H3K9me at promoters of growth-promoting genes is critical feature of cellular senescence and terminal differentiation associated with gene repression (Narita et al., 2003; Kotake et al., 2007; Sdek et al., 2011). However, it is not clear if H3K9me2 and H3K9me3 are equally important in gene silencing of postmitotic cells. Establishment of H3K9me2 and H3K9me3 requires different methyltransferases (G9a/GLP and Suv39h1/2 respectively), and the nuclear sublocalization of these two modifications in postmitotic cells is different. H3K9me2 is found in both euchromatin and heterochromatin regions while H3K9me3 is exclusively co-localized with heterochromatin (Sdek et al., 2011). This finding suggests H3K9me2 and H3K9me3 have slightly different roles in the repression of gene expression. Since Rb is intimately involved in targeting these methylations, knocking out Rb family member expression in specific cell types allows dissection of the roles of H3K9me2 and H3K9me3 in terminally differentiated cells (Sdek et al., 2011). Acute depletion of Rb alone does not trigger cell cycle reentry in ACMs although it dramatically reduces H3K9me2 levels, indicating H3K9me2 is dispensable for maintenance of the postmitotic state in ACMs (Sdek et al., 2011). In contrast, deleting both Rb and p130 disrupts heterochromatin and allows cell cycle reentry in ACMs; however, H3K9me3 levels were unchanged (Sdek et al., 2011), which suggests H3K9me3 is established and maintained by an Rb-independent pathway and the presence of H3K9me3 alone is not sufficient for heterochromatin formation in CMs. In contrast *in vitro* experiments have shown that knockdown of Suv39h1 resulted in de-suppression of cell cycle gene and terminal differentiation failure in myocytes, suggesting that H3K9me3 is, at least, necessary for establishing myocyte terminal differentiation (Ait-Si-Ali et al., 2004; Sdek et al., 2011).

### H3K9me2/3 MEDIATOR HP1

The ability of Rb to stably repress transcription is related to its capacity to recruit HP1s to target gene promoters resulting in their incorporation into heterochromatin (Nielsen et al., 2001; Narita et al., 2003). HP1 is a family of proteins (HP1α, -β, and -γ) that play an important role in gene silencing in many organisms (James and Elgin, 1986; Kellum, 2003) by establishing and maintaining heterochromatin (Daniel et al., 2005). HP1 family members typically differ in their subcellular localization and interaction partners and thus likely have distinct cellular functions (Minc et al., 1999, 2001; Auth et al., 2006). A p130 and HP1α complex is recruited to E2F regulated promoters during neuronal differentiation to induce cell cycle exit (Panteleeva et al., 2007). Rb directly promotes permanent cell cycle exit in senescent cells by recruiting HP1γ to E2F responsive promoters that have undergone methylation of H3K9 (Narita et al., 2003). The role of HP1s in the heart largely remains unknown. All three HP1 family members are expressed in ACMs although their subnuclear localization differs. HP1γ in particular is essential for stably repressing proliferation-promoting genes in ACMs, and although HP1 family members share similar structure, HP1α and HP1β could not compensate for the loss of function of HP1γ in CMs (Sdek et al., 2011). HP1γ binds to G2/M and cytokinesis gene promoters in ACMs but disassociated from these promoters when Rb/p130 were acutely deleted, although H3K9me3 levels at G2/M and cytokinesis gene promoters remained intact. The dissociation of HP1γ at G2/M and cytokinesis gene promoters correlated with loss of heterochromatin in ACMs’ nuclei, re-expression of G2/M and cytokinesis genes as well as cell cycle re-entry. Given the important role of HP1s in heterochromatin formation, the absence of HP1γ recruitment appeared to be the key factor in the disruption of heterochromatin and the reinduction of proliferation capacity in ACMs lacking Rb/p130. Thus, in CM terminal differentiation, Rb and p130 serve as a bridge to link histone modifications and heterochromatin formation through their interaction with HP1γ. Heterochromatin stably represses the expression of proliferation-promoting genes and maintains the postmitotic phenotype of ACMs (Sdek et al., 2011; **Figure 1C**).

### H3K27 METHYLATION

The role of H3K27me3 in terminal differentiation is not clear although the promoters of proliferation related genes displayed higher levels of H3K27me3 in ACMs and skeletal myotubes (Blais et al., 2007; Sdek et al., 2011). H3K27me3 is important for stable gene repression, including suppression of E2F-dependent genes, in certain contexts (Blais et al., 2007). ACMs from mice where Rb and p130 were deleted embryonically did not undergo permanent cell cycle exit (MacLellan et al., 2005). H3K27me3 and heterochromatin formation in these CMs were dramatically impaired although global H3K9me3 levels were unchanged (Sdek et al., 2011). H3K27me3 is not exclusively enriched on heterochromatin, indicating H3K27me3 might relate to early stages of heterochromatin formation and repression of genes required for proliferation.

## Rb REGULATION OF EPIGENETIC GENE SILENCING

Rb is the prototypical member of a gene family encoding three structurally and functionally similar proteins, Rb, p107, and p130 (Chen et al., 1996). Rb also plays critical roles in senescence and terminal differentiation associated with irreversible growth arrest including CMs (Narita et al., 2003; Sdek et al., 2011). Rb mediated inhibition of gene expression can be achieved by two mechanisms: direct inhibition of E2F or recruitment of epigenetic remodeling factors (Gonzalo and Blasco, 2005). These two mechanisms are selective: some promoters are repressed by the first mechanism, whereas other promoters, particularly cell cycle genes, are silenced by the second mechanism (Brehm et al., 1998; Luo et al., 1998; Angus et al., 2004). Rb family members associate with multiple chromatin remodeling factors, including HDACs (Luo et al., 1998; Magnaghi-Jaulin et al., 1998), Suv39h1 (Nielsen et al., 2001), HP1 (Nielsen et al., 2001), Ezh2 (Tonini et al., 2004), Pc2 (Dahiya et al., 2001), and DMNT1 (Robertson et al., 2000). Thus Rb affects a wide range of epigenetic regulation pathways, including histone acetylation, histone methylation and DNA methylation.

The major effect of Rb on the histone acetylation pathway is facilitating deacetylation of target gene promoters by recruiting HDACs (Brehm et al., 1998; Luo et al., 1998; Magnaghi-Jaulin et al., 1998). Class I HDACs (HDAC1, -2, and -3) directly interact with Rb through the pocket domain (Lai et al., 1999). Similar to many Rb-interacting proteins, HDAC1 contain an leucine-X-cysteine-X-glutamic acid, X stans for any any amino acid (LXCXE) motif, which allows direct interaction with Rb (Magnaghi-Jaulin et al., 1998). Recruitment of HDAC1 to E2F-regulated promoters is important for Rb-mediated S phase repression (Brehm et al., 1998). Rb also regulates H3K9me3, which is important for gene silencing and heterochromatin formation. Histone methltransferase, Suv39h1/2, is specifically required for H3K9me3 establishment and maintenance (Schotta et al., 2004; Siddiqui et al., 2007). Rb physically interacts with Suv39h1 and it has been suggested that this interaction is critical for Suv39h1’s gene suppression activity (Nielsen et al., 2001). Recent studies, however, have demonstrated that global levels of H3K9me3 is normal in fibroblasts that are triply deficient for Rb, p107 and p130 (Gonzalo et al., 2005; Siddiqui et al., 2007), indicating that Rb family members are dispensable for Suv39h1 imposed H3K9me3. H3K9me3 is specifically recognized by HP1 protein, and the binding of HP1 protein at H3K9me3 site is important to transmit its biological signals (Bannister et al., 2001; Lachner et al., 2001; Nakayama et al., 2001). HP1 also recruits Suv39h1 and propagates H3K9me3 to adjacent chromatin (Yamamoto and Sonoda, 2003; Hathaway et al., 2012). Thus, heterochromatin formation is a self-assembling framework of “tethers” (H3K9me3) and “adaptors” (HP1) where the HP1 molecules bound to neighboring nucleosomes dimerize through their chromoshadow domains, leading to HP1-nucleosome complexes and chromatin condensation (Breitbart et al., 1985; Hathaway et al., 2012). HP1s contain an LXCXE (or LXCXD) motif which allows interaction with both Rb and p130 (Nielsen et al., 2001; Panteleeva et al., 2007); thus RB can directly target HP1 on cell cycle gene promoters (Nielsen et al., 2001; Panteleeva et al., 2007; Sdek et al., 2011), which seems to be a key step for the initiation of terminally differentiation and senescence. These findings are supported by fact that the absence of Rb results in loss of heterochromatin and disrupted H3K9me3 nuclear distribution, even though global H3K9me3 is intact (Narita et al., 2003; Gonzalo et al., 2005; Sdek et al., 2011; Zhang et al., 2013).

It has been demonstrated that Rb is also required for establishment and maintenance of H3K27me3 (Blais et al., 2007; Kotake et al., 2007). Rb physically interacts with PRC2, the major enzyme complex that methylates H3K27. In growing human and mouse primary cells, expression of p16, a prominent cell cycle inhibitor, is repressed by H3K27me3 at the p16 locus, which is established by PRC2. This recruitment of PRC2 is Rb dependent (Kotake et al., 2007). In contrast Rb also interacts with Pc2, the effector protein of the PCR1 complex that recognizes and binds to H3K27me3 (Dahiya et al., 2001; Cao et al., 2002); it has been reported that Pc2 cooperates with Rb to inhibit expression of cyclin A and cdc2 in senescence cells (Dahiya et al., 2001). The mechanism underlying this function switching of Rb remains to be elucidated.

Importance of Rb on CM terminal differentiation through epigenetic mechanism was demonstrated (Sdek et al., 2011). Although cardiac-specific information is limited, epigenetic role of Rb has been demonstrated in senescence and cell line model. Potentially the similar mechanisms discussed above are mediating CM terminal differentiation.

## CONCLUSION

Commitment to a particular lineage requires both the repression of unnecessary genes while simultaneously up-regulating lineage-specific genes. High-throughput DNA sequencing technology has enable the search for the binding sites of cardiac TFs, enhancers, epigenetic marked histones and chromatin modifying factors on a genome-wide level by ChIP-sequencing (Blow et al., 2010; He et al., 2011; Paige et al., 2012; Wamstad et al., 2012; Papait et al., 2013). Core cardiac TFs such as Nkx2.5, Mef2c, GATA4, Tbx5, and SRF and cell cycle master regulator RB/p130 have been shown to form complexes with epigenetic modifying proteins and these complex multimers lead to modifications of histones at promoters of cardiac and cell cycle genes which locks in the ACM phenotype. This review has attempted to summarize the advances that have been made in our understanding of epigenetic regulation in cardiac differentiation and development. Although increasing evidence suggests crucial roles of epigenetic modifying proteins and epigenetic marks, their specific function in cardiac lineage commitment and differentiation as well as their orchestrating mechanisms still remain to be elucidated. Regardless, understanding this epigenetic regulation will undoubtable uncover new insights into cardiovascular biology and potentially facilitate development of novel targets for cardiovascular therapeutics and regeneration.

## Conflict of Interest Statement

The authors declare that the research was conducted in the absence of any commercial or financial relationships that could be construed as a potential conflict of interest.

## Abbreviations

ACMs: adult cardiac myocytes
CBP: cAMP response element binding protein-binding-protein
CDK: cyclin dependent kinase
CGIs: CpG islands
CMs: cardiac myocytes
CpG: cytosine-guanine dinucleotides
DNMT: DNA methyltransferase
HAT: histone acetyltransferase
HDAC: histone deacetylase
HDACi: HDAC inhibitor
HDM: histone demethylases
HMT: histone methyltransferase
HP1: heterochromatin protein 1
jmj: jumonji
KO: knockout
Rb: retinoblastoma
TF: transcription factor
TSA: Tricostatin A
WHSC1: Wolf-Hirschhorn Syndrome Candidate 1.
